# A case report on transient global ventricular wall thickening secondary to acute myocarditis: Focus on the unique role of cardiac MRI

**DOI:** 10.1097/MD.0000000000019223

**Published:** 2020-02-21

**Authors:** Tiantian Xu, Hongjie Hu

**Affiliations:** Department of Radiology, The Sir Run Run Shaw Hospital, Zhejiang University School of Medicine, Hangzhou, Zhejiang Province, China.

**Keywords:** cardiac magnetic resonance imaging, myocarditis, ventricular wall thickening

## Abstract

**Introduction:**

Transient left ventricular wall thickening is known to develop in the acute phase of myocarditis, with several reports documenting this unusual mode of myocarditis. Diagnosing myocarditis can be challenging because symptoms, clinical exam findings, electrocardiogram results, biomarkers, and echocardiogram results are often non-specific. Therefore, cardiac magnetic resonance imaging has become the primary non-invasive imaging tool in patients with suspected myocarditis.

**Patient concerns and diagnosis:**

A 51-year-old male was referred to our hospital with a 20-day history of fever. Initial echocardiogram demonstrated diffuse concentric left ventricular hypertrophy with depressed left ventricular diastolic function, previously misdiagnosed as restrictive cardiomyopathy. Cardiac magnetic resonance imaging (MRI) showed global ventricular wall thickening, and the negative delayed enhancement made hypertrophic cardiomyopathy and myocardial amyloidosis less likely. This information, along with laboratory analyses, led to a diagnosis of acute myocarditis.

**Interventions and outcomes:**

The patient underwent a treatment regimen, including a prescription of levofloxacin and other supporting treatments. During the period following, the patient experienced a few minor episodes of atypical chest pain with spontaneous remission. The patient was discharged after 8 days of hospitalization. A cardiac MRI evaluation was repeated after 17 months, this time showing that the wall thickness had returned to normal; the myocarditis resolved without sequela.

**Conclusions:**

In summary, we report on a case of transient global ventricular wall thickening secondary to acute myocarditis, which rarely has been described previously. Our study demonstrates that transient ventricular wall thickening related to myocardial interstitial edema also can involve the right ventricular wall, a fact that is important in diagnosis and differential diagnosis. Cardiovascular magnetic resonance currently is considered the most comprehensive and accurate diagnostic tool in patients with suspected myocarditis.

## 1 Introduction

Transient left ventricular (LV) wall thickening is known to develop in the acute phase of myocarditis. Several reports^[1–5]^ have investigated this unusual mode of myocarditis. A large number of endomyocardial biopsies from myocarditis patients in both the acute and convalescent phases demonstrate that this wall thickening results from the interstitial edema that accompanies myocarditis inflammation.^[4]^ Diagnosing myocarditis can be challenging as its symptoms, clinical exam findings, electrocardiogram (ECG) results, biomarkers, and echocardiogram data are often non-specific. Endomyocardial biopsy (EMB) is an established method for diagnosing myocarditis, but carries the risk of complications and false negative results. Therefore, cardiac magnetic resonance imaging (MRI) has become the primary non-invasive imaging tool in patients with suspected myocarditis.

To the best of our knowledge, the body of literature has little documentation of global ventricular wall thickening during the acute stage of myocarditis. We report this case to emphasize the unique role and differential diagnostic value of cardiac MRI, which is the only non-invasive modality currently available for evaluating the temporal evolution of myocardial involvement after acute myocarditis.

## 2 Case report

**Figure 1:**
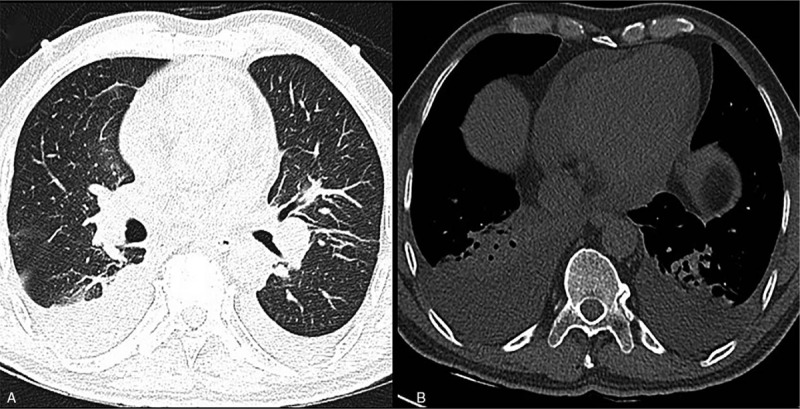
(A: lung window) No obvious effusion was observed in either lung; (B: mediastinal window) bilateral pleural effusion with bilateral partial atelectasis and pericardial effusion.

A 51-year-old male presented to the emergency department complaining of fever lasting for 20 days, shortness of breath, chest tightness, and a cough spanning 4 days. He had no significant past medical history. His body temperature was 37.5°C, his blood pressure was 102/76 mm Hg, his heart rate was 109 beats per minute, and his physical examination was generally normal. Laboratory studies showed a white blood cell (WBC) count of 14.9 × 10^9^/L (reference range, 3.5–9.5 × 10^9^/L). Results also indicated troponin T to be 5.26 ng/mL (reference range, 0–0.11 ng/mL); creatine kinase (CK) 300 IU/L (reference range, 24–194 IU/L); creatine kinase enzymes (CKMB) 33 IU/L (reference range, 0–24 IU/L); and C-reactive protein (CRP) 9.1 mg/L (reference range, 0.0–5.0 mg/L). Chest computed tomography (CT) showed a moderate amount of pleural effusion with bilateral partial atelectasis and pericardial effusion (Fig. 1). ECG on admission revealed sinus tachycardia, critical low voltage of limb lead, mild depression of the ST segment of the anterior wall lead, and a prolonged critical QT interval. Transthoracic echocardiography showed diffuse concentric LV hypertrophy with depressed LV diastolic function and a small pericardial effusion, which had been misdiagnosed as restrictive cardiomyopathy. The patient subsequently underwent coronary angiography, which demonstrated normal coronary anatomy with a right dominant system.

**Figure 2:**
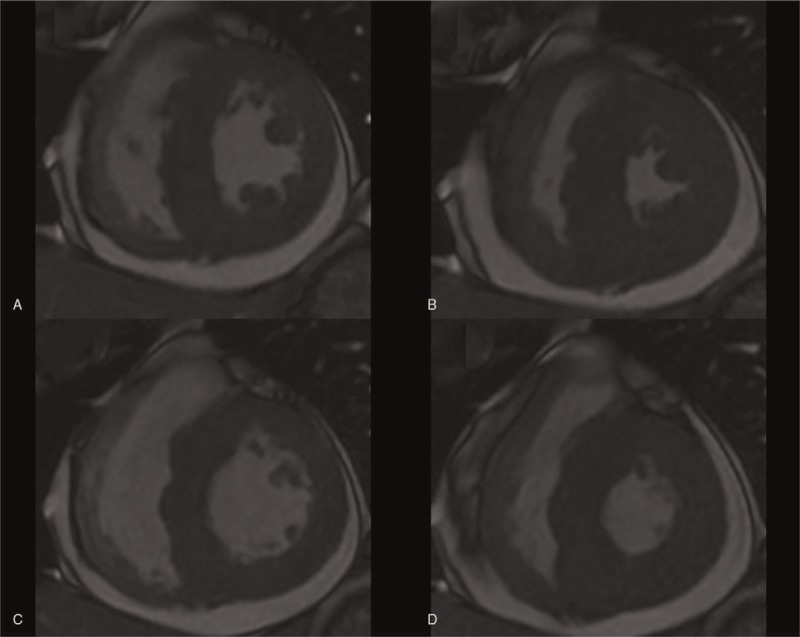
Cine images during end-diastolic (A and C) and end-systolic (B and D) phases demonstrated global ventricular wall thickening (left ventricular wall thickness including septal wall = 13–17 mm and right ventricular wall thickness = 7–8 mm) in the short axis at the level of the central (A and B) and basal ventricles (C and D). The pericardial effusion was existent, with a normal left ventrical ejection fraction of 71%. The normal thickness of the left ventricular wall, including septal wall ≤ 12 mm and right ventricular wall, had a thickness ≤ 5 mm.

**Figure 3:**
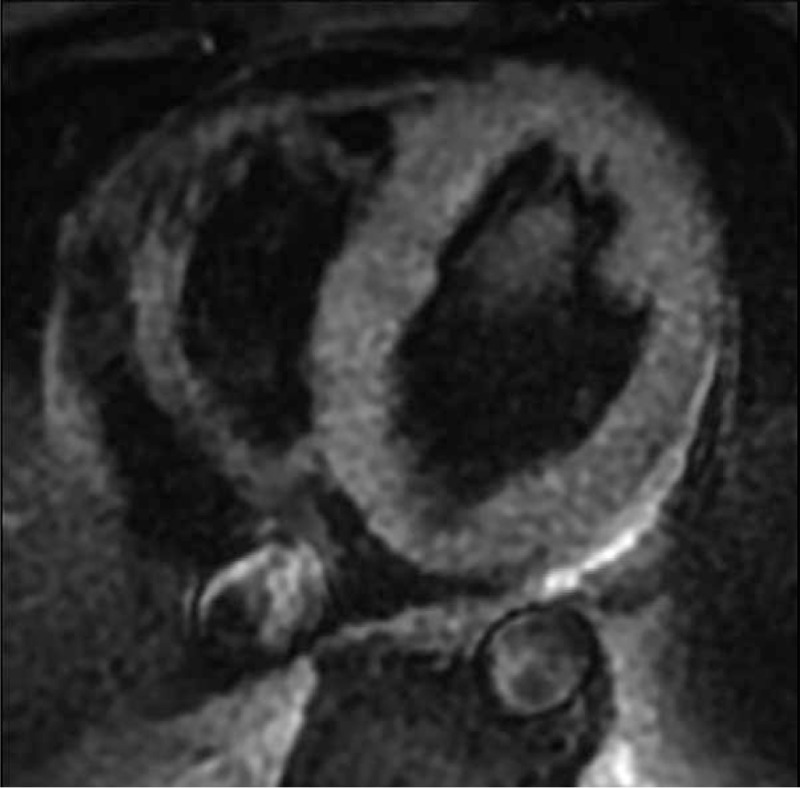
T2-weighted STIR images of transverse section showed no significant high signal (myocardial edema) of the ventricle wall.

**Figure 4:**
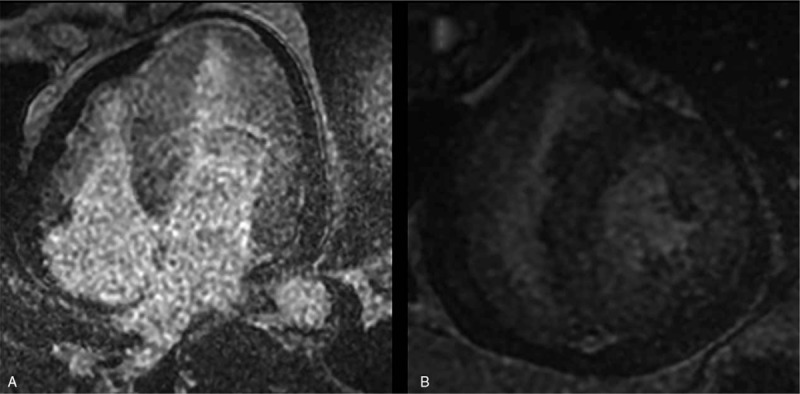
There was no myocardial area of late post-contrast enhancement detected on images in the four-chamber view (A) and short axis (B). ∗STIR = short time inversion recovery.

**Figure 5:**
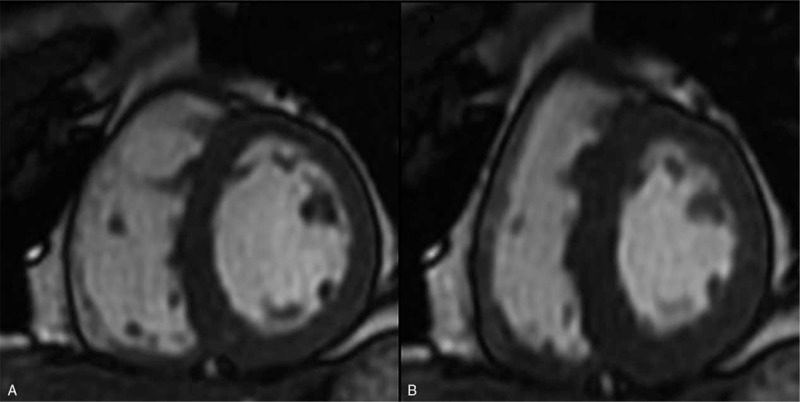
(A repeated MRI after 17 months): Cine images during end-diastolic (A) and end-systolic (B) phases demonstrated global normal ventricular wall thickness (left ventricular wall thickness, including septal wall, was 8–12 mm, and right ventricular wall thickness was 4–5 mm) in the short axis at the level of the central ventricles. The pericardial effusion was absorbed completely, with a normal left ventrical ejection fraction of 79%. ^∗^MRI = magnetic resonance imaging.

**Figure 6:**
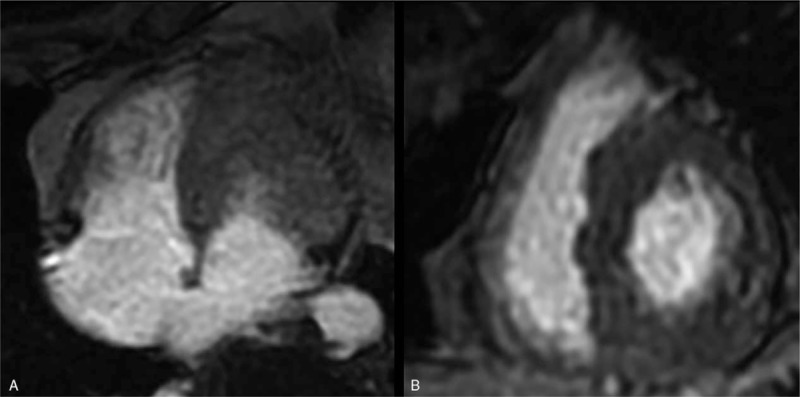
(A repeated MRI after 17 months): There was no myocardial area of late post-contrast enhancement detected on images in the four-chamber view (A) and short axis (B). ^∗^MRI = magnetic resonance imaging.

Cardiac magnetic resonance imaging then was performed with a 1.5 T GE (GE Signa HD excite, America). Cine images were acquired using a steady state free precession sequence, revealing global ventricular hypertrophy with normal regional and global systolic function, accompanied by pericardial effusion (Fig. 2). T2-weighted short-time inversion recovery (STIR) images showed no significant high signal (myocardial edema) in the ventricle wall (Fig. 3). There was also no myocardial area of late post-contrast enhancement (myocardial fibrosis/necrosis) detected on the images acquired with a T1-weighted segmented inversion-recovery gradient echo sequence starting 10 min after an intravenous bolus of 0.15 mmol/kg gadobutrol (Fig. 4). Based on the Lake Louise Criteria (LLC) proposed by the International Consensus Group on Cardiovascular Magnetic Resonance in Myocarditis,^[6]^ a diagnosis of acute myocarditis was difficult to make, but it was also difficult to rule out this diagnosis. The negative delayed enhancement in MRI made a diagnosis of hypertrophic cardiomyopathy (HCM) and myocardial amyloidosis less likely; based on the laboratory analyses, we finally made a clinical diagnosis of acute myocarditis. EMB was not performed. The patient underwent a treatment regimen including a prescription of levofloxacin and other supporting treatments. During the follow-up period, the patient experienced a few minor episodes of atypical chest pain with spontaneous remission. The patient was discharged after 8 days of hospitalization. A cardiac MRI evaluation was repeated after 17 months, this time showing that the wall thickness had returned to normal (Figs. 5 and 6). The myocarditis resolved without sequelae.

## 3 Discussion

Acute myocarditis is defined as an inflammatory disease of the myocardium that can result from a wide variety of infectious agents (viruses, bacteria, and others), systemic diseases, drugs, and toxins.^[7]^ Clinically, myocarditis can manifest as acute heart failure, ventricular arrhythmias, or cardiogenic shock. It is associated with substantial morbidity and mortality. Children diagnosed with acute myocarditis have only a 60% likelihood of transplantation-free survival at 10 years. Myocarditis has been linked to sudden cardiac death in young adults in up to 12% of cases and identified as the underlying etiology of dilated and arrhythmogenic right ventricular cardiomyopathy.^[8]^ The true incidence of myocarditis is unknown due to varying clinical presentations and infrequent diagnostic testing. Diagnosis of this condition is difficult because the symptoms, clinical exam findings, ECG results, biomarkers, and echocardiogram results are non-specific.

In medical practice, physicians rely on a combination of clinical features, laboratory analyses, and imaging results to diagnose myocarditis. EMB allows a definitive diagnosis, but it is not very sensitive and is highly invasive and costly. Current guidelines promote its use only in patients with acute unexplained heart failure complicated by hemodynamic instability.^[9]^ Cardiac magnetic resonance imaging (CMR) has recently become the primary non-invasive imaging tool for patients with suspected myocarditis.^[6]^ The diagnosis of myocarditis by cardiac MRI depends on meeting at least two of the three LLC,^[6]^ consisting of tissue characterization findings including edema, hyperemia, and necrosis. Based on pooled data with clinical and histologic validation from the International Consensus Group on Cardiovascular Magnetic Resonance in Myocarditis, receiving two out of three positives on these LLC criteria yields a sensitivity of 67% and a specificity of 91%.^[10]^ However, the case presented here did not meet the LLC, so the clinical diagnosis of acute myocarditis was made based on the history of a 20-day fever, significant elevation of cardiac-specific enzymes, and MRI changes. The diagnosis was further confirmed by the favorable course of the disease with complete resolution of clinical signs and symptoms, as well as the wall thickness returning to normal, as shown by MRI during follow-up.

Several case reports have described thickening of the LV wall during the acute stage of myocarditis. Autopsy indicates that this thickening occurs ∼3 weeks after the acute illness.^[11]^ Furthermore, this wall thickening shows near-normalization after days 6 to 8, indicating a transient finding limited to the acute phase.^[11]^ The transient ventricular wall thickening that occurs in the acute stage of myocarditis results from myocardial interstitial edema.^[4]^ However, the global ventricular wall thickening during the acute stage of myocarditis has rarely been described. We believe that this case demonstrates that the right ventricular wall also can be involved in myocarditis. Wall thickening due to this interstitial edema mostly results in concentric hypertrophy. Occasionally, it may also cause asymmetrical septal hypertrophy and narrowing of the cavity of the left ventricle (LV), producing hypertrophic obstructive cardiomyopathy.^[12]^ In diagnosis and differential diagnosis, it is important to note that when such concentric hypertrophy is present, the LV end-diastolic volume decreases and systolic function declines, both of which induce a reduction in stroke volume. When such concentric hypertrophy is present, the LV end-diastolic volume decreases, and systolic function declines, both of which reduce stroke volume. Myocardial edema is one of the main features of the human inflammatory response in acute myocarditis. As this kind of marked transient wall thickening is unlikely to develop in conditions other than acute myocarditis, it should be recognized as a key characteristic of myocarditis in the acute phase.^[13]^

In our case, echocardiogram led to a misdiagnosis of restrictive cardiomyopathy. ECG holds a low sensitivity and specificity in the diagnosis of myocarditis, limiting its role in differential diagnosis. CMR is able to confirm an acute myocarditis diagnosis in the majority of patients suspected to have the condition based on clinical criteria. Nonetheless, it faces some limitations in diagnosing myocarditis. For instance, interpreting MRI images can be particularly challenging when there is global myocardial involvement. Detecting edema on conventional T2 weighted imaging (T2WI) may be restricted by image quality and objective image interpretation, and global edema might be missed when the skeletal muscle is also inflamed. Global myocardial enhancement may also be confusing on late gadalinum enhancement (LGE) images due to incomplete myocardial nulling; conversely, it may be ignored due to diffuse myocardial nulling.^[14]^ T1, T2, and ECV (extracellular volume fraction) mapping are novel CMR techniques for quantitative tissue characterization with tight normal ranges, allowing a more objective assessment of myocardial tissue properties. Importantly, parametric mapping techniques appear to overcome some of the aforementioned technical limitations of the CMR while enabling the assessment of diffuse myocardial injury. They are highly sensitive to increased free water content, rendering them ideal for detecting acute myocardial inflammation/edema.^[15]^ Additionally, CMR data play an important role not only for diagnosis but also for prognosis of myocarditis. Currently, there are few studies supporting the prediction that LGE 4 weeks after onset of symptoms is predictive for functional and clinical long-term outcomes, nor is there much research asserting that relatively early CMR findings may predict longer-term outcomes.^[16]^

In our case, in MRI, the T2-weighted STIR images showed no significant high signal (myocardial edema) of the ventricular wall. Also, there was no significant delayed enhancement (myocardial fibrosis/necrosis) detected, due to the poor image quality from a low signal-to-noise ratio. Unfortunately, novel mapping techniques were not available in our hospital. In this situation, we lacked sufficient imaging support to make a diagnosis of myocarditis, but we also could not exclude the diagnosis. The global ventricular wall thickening in our case, however, pointed to global myocardial involvement myocarditis. Diffuse myocardial inflammation and intracellular and interstitial edema can lead to insufficient regional redistribution of gadolinium, which results in a normal LGE image. Abundant interstitial edema and faster water exchange in cells with dysfunctional membranes cause magnetization transfer from the extracellular gadolinium to the intracellular water, further reducing the contrast. Similarly, appreciation of the increased T2 signal intensity relies on regional differences. The edema ratio is based on the relative comparison of signal to skeletal muscle. This signal also can be affected, thus resulting in a pseudo-normalized value.^[17]^ Furthermore, the presence of a pericardial effusion was also suggestive of myocarditis.

In clinical work, the differential diagnosis of myocarditis is very important. When patients present with acute chest pain, abnormal ECG, positive cardiac enzymes, and normal coronary arteries, cardiac MRI has demonstrated the ability to differentiate between cases of acute myocarditis and other clinical conditions, including acute myocardial infarction. This ability rests on the capacity of cardiac MRI to identify specific patterns of myocardial damage (i.e., subepicardial/intramyocardial distribution in myocarditis versus subendocardial/transmural distribution in acute myocardial infarction). Interestingly, the evolution of MRI patterns over time observed in myocarditis can be useful in monitoring the disease course, and, potentially, in defining the correct management strategy, as demonstrated by a previous single case study.^[13]^

Myocardial wall thickness depends on 3 elements: the number of myocytes, myocyte size, and the volume of the interstitial space.^[18,19]^ In HCM, the increased left ventricular wall thickness (LVWT) is mediated by an increased mass in individual myocytes and interstitial fibrous connective tissue, with the former being the most important component.^[18]^ Other diseases can mimic HCM by causing an increase in LVWT that is not due to myocyte hypertrophy or hyperplasia; these conditions include intracellular accumulation of metabolic products (storage diseases) or interstitial infiltration of proteins, cells or fluid, such as in amyloidosis or myocarditis.^[4]^ Thus, myocarditis presenting as ventricular thickening requires differential diagnosis from HCM and myocardial amyloidosis. Cardiac magnetic resonance imaging results are helpful in providing non-invasive evidence to demonstrate whether the LV thickening is related to myocardial edema or myocardial hypertrophy, a distinction echocardiography cannot make. More importantly, different delayed enhancement patterns play a key role in distinguishing between myocarditis and other lesions that can cause cardiac hypertrophy, like HCM and myocardial amyloidosis.^[20]^ Transient myocardial thickening can then potentially result from emotional or physical stress associated with the reported antecedent events.^[21]^ Takotsubo cardiomyopathy is a transient LV dysfunction most frequently characterized by an apical “ballooning” phenotype in people, but myocardial edema mimicking HCM and myocarditis also has been described in Takotsubo cases^[22,23]^; as such differentiation from true HCM and myocarditis requires advanced cross-sectional imaging.

Although CMR is a promising technology, its sensitivity in diagnosing myocarditis must be improved, and additional imaging techniques should be explored in order to acquire more detailed information regarding both the mechanism and the etiology of myocardial inflammation. Future research potentially could focus on developing novel imaging agents that highlight areas of active myocarditis on CMR. An alternative approach would be to develop molecular labels for CMR-based virus detection.

In summary, we have reported on a case of transient global ventricular wall thickening secondary to acute myocarditis, a condition rarely described in previous work. This report seeks to demonstrate that the transient ventricular wall thickening related to myocardial interstitial edema can also involve the right ventricular wall, a fact that is important in both diagnosis and differential diagnosis. Cardiovascular magnetic resonance (CMR) currently is considered the most comprehensive and accurate diagnostic tool in patients with suspected myocarditis. Novel CMR mapping techniques provide high diagnostic accuracy when diagnosing acute myocarditis; they may become promising successors of LLC's classic elements for routine diagnostic protocols. Although CMR has high sensitivity and specificity for myocarditis, advances are needed to assist in determining the underlying cause of the condition; such progress would increase the value of such techniques while guiding targeted interventions.

## Author contributions

**Supervision:** Hongjie Hu.

**Writing – original draft:** Tiantian Xu.

**Writing – review & editing:** Tiantian Xu.
